# Correlation of [^18^F] FDG-PET/CT with dosimetry data: recurrence pattern after radiotherapy for head and neck carcinoma

**DOI:** 10.1186/s13014-021-01787-5

**Published:** 2021-03-21

**Authors:** C. Pisani, L. Vigna, F. Mastroleo, G. Loi, V. Amisano, L. Masini, L. Deantonio, P. Aluffi Valletti, G. Sacchetti, M. Krengli

**Affiliations:** 1 Division of Radiation Oncology, University Hospital Maggiore della Carità, Novara, Italy; 2 Department of Translational Medicine, University of “Piemonte Orientale”, Via Solaroli, 17, 28100 Novara, Italy; 3 Service of Medical Physics, University Hospital Maggiore della Carità, Novara, Italy; 4 Division of ENT, University Hospital Maggiore della Carità, Novara, Italy; 5 Division of Nuclear Medicine, University Hospital Maggiore della Carità, Novara, Italy

**Keywords:** Head and neck cancer, Radiotherapy, Recurrence, FDG-PET/CT, MTV

## Abstract

**Objective:**

To analyze the pattern of failure in relation to pre-treatment [^18^F] FDG-PET/CT uptake in head and neck squamous cell carcinoma (HNSCC) patients treated with definitive radio-chemotherapy (RT-CHT).

**Methods and materials:**

From 2012 to 2016, 87 HNSCC patients treated with definitive RT-CHT, with intensity modulated radiation therapy with simultaneous integrated boost, underwent pre-treatment [^18^F] FDG-PET/CT (PET_pre_), and MRI/CT for radiotherapy (RT) planning purposes. Patients with local recurrence, received [^18^F] FDG-PET/CT, (PET_rec_) at the time of the discovery of recurrence. In these patients, the metabolic target volume (MTV), MTV_pre_ and MTV_rec_ were segmented on PET images by means of an adaptive thresholding algorithm. The overlapping volume between MTV_pre_ and MTV_rec_ (MTV_pre&rec_) was generated and the dose coverage of MTV_rec_ and MTV_pre&rec_ was checked on the planning CT using the D99 and D95 dose metrics. The recurrent volume was defined as: ‘‘In-Field (IF)’’, “Marginal recurrence” or ‘‘Out-of-Field (OF)’’ if D95 was respectively equal or higher than 95%, D95 was between 95 and 20% or the D95 was less than 20% of prescribed dose.

**Results:**

We found 10/87 patients (11.5%) who had recurrence at primary site. Mean MTV_pre_ was 12.2 cc (4.6–28.9 cc), while the mean MTV_rec_ was 4.3 cc (1.1–12.7 cc). Two recurrences resulted 100% inside MTV_pre_, 4 recurrences were mostly inside (61–91%) and 4 recurrences were marginal to MTV_pre_ (1–33%). At dosimetric analysis, five recurrences (50%) were IF, 4 (40%) marginal and one (10%) OF. The mean D99 of the overlapping volumes MTV_pre&rec_ was 68.1 Gy (66.5–69.2 Gy), considering a prescription dose of 70 Gy to the planning target volume (PTV).

**Conclusion:**

Our study shows that the recurrence may originate from the volume with the highest FDG-signal. Tumor relapse in the high-dose volume support the hypothesis that an intensification of the dose on these volumes could be further assessed to prevent local relapse.

## Introduction

Head and neck cancers (HNCs) represent the 6th most common cancer in the world and 5% of malignancies worldwide [1]. Approximately 60% of patients are in locally advanced stage at diagnosis [2] and the standard of care includes radiation therapy (RT), besides surgery, with curative and organ preservation [3].

Despite improvements in RT planning and techniques, recurrences remain the greatest obstacle to long-term survival in HNC patients [4]. When feasible, surgery after tumor recurrence is the first-choice treatment, although re-irradiation could also be considered in selected patients. However, recurrent HNCs occurring in a previously irradiated volume have a poor prognosis and therapeutic decisions remain controversial [4].

According to the current literature, only few studies describe RT failure in terms of localization and biological features. Chao et al. observed that almost 50% of failures after definitive RT were infield and occurred within the 95% isodose coverage region, despite an adequate target definition and coverage [5].In addition to CT and MRI, [^18^F] FDG-PET/CT can be used to assess metabolic tumor function and anatomic localization for staging, RT planning, and restaging during follow-up in HNC [6–8]. Some authors found that the majority of HNC failures map to the pre-treatment PET abnormality [9–11]. These studies suggest the presence of radioresistant tumor sub-volumes within the primary target, that could be detected by [^18^F] FDG-PET/CT. In such cases, metabolic imaging could drive tailored treatment intensification by dose painting.

In this study, we analyzed the recurrence pattern of HNC patients treated with definitive radio-chemotherapy (RT-CHT) in relation to pre-treatment [^18^F] FDG-PET/CT uptake. We contoured and compared the metabolic target volume (MTV) on both pre-treatment and at-relapse assessments, considering the isodoses of the original treatment plan to analyze the dose coverage of these MTVs.

## Methods and materials

### Patient selection

We retrospectively analyzed patients from 2012 to 2016, affected by HNC and treated with definitive RT or RT-CHT. The study was performed following the rules of our Institution, after review board consensus.

Selection criteria for study purposes included adult age, squamous cell histology, availability of pre-treatment PET/CT, RT (± concomitant chemotherapy) to a curative dose by intensity modulated radiation therapy (IMRT) and follow-up longer than 2 years. We excluded patients with salivary gland or nasal/paranasal sinus tumors, neck metastases from occult primary tumor, history of previous cancer, previous surgery in head and neck region and neoadjuvant/adjuvant chemotherapy and radiotherapy to non-curative dose. Furthermore, all cases had to be discussed by a multidisciplinary board at diagnosis and at the time of tumor relapse.

### Primary treatment and follow-up

Treatment position for primary RT was defined at planning CT-scan: patients were supine with neck support and customized thermoplastic mask. Planning CT was performed with contiguous slices of 3 mm thickness from the vertex to the aortic arc. Pre-treatment PET/CT (PET_pre_) was performed by hybrid PET/CT scanner (Biograph 16 HI-REZ PET/TC, Siemens Medical Solutions) in treatment position from the proximal femur to the base of the skull. PET_pre_ was performed within 5 working days from planning CT. Volumes of interest were contoured by an expert radiation oncologist and checked by a second experienced radiation oncologist reaching a consensus on the following volumes: the gross target volume (GTV), using CT and MRI images and clinical examination, the high risk clinical target volume (HR-CTV), including GTV considering the microscopic diffusion viable in the three dimensions but almost > 0.5 cm, the low risk clinical target volume (LR-CTV), encompassing elective nodes on the basis of the location of the primary tumor. The planning target volume (PTV) was determined geometrically expanding by 4 mm the corresponding CTV.

Each patient was treated by IMRT with simultaneous integrated boost (IMRT-SIB) using 5–8 coplanar and non-coplanar 6 MV photon beams. Prescribed doses were 70 Gy to HR-PTV-T, 66 Gy to HR-PTV-N, 60 Gy to Intermediate-PTV, 54 Gy to LR-PTV with conventional fractionation. Dose was prescribed to PTV to ensure the required coverage of the planning target volume with the 95% isodose line. IMRT-SIB was delivered by linear accelerator with position verified by weekly Cone Beam CT/portal imaging.

All patients had systematic clinical and imaging follow-up, performed every 3 months during the first two years, then every 6 months over 3–5 years from the date of the treatment and annually thereafter. Post-treatment baseline imaging (contrast enhanced CT, MRI and PET/CT) was performed 3 months after the end of treatment. Radiologic assessment of distant metastases (thoracic CT, liver ultrasound) was thereafter performed by physical examination.

### Imaging acquisition and MTV definition at recurrence

Patients developing local recurrence at the site of the primary tumor underwent PET/CT (PET_rec_) performed with the same hybrid PET/CT scanner and by the same procedure used for PET_pre_.

In these patients, MTV was delineated by an Adaptive Threshold Algorithm (ATA) on both PET_pre_ and PET_rec_ thus obtaining a MTV_pre_ and a MTV_rec_, respectively. Only primary tumor and local recurrence was considered for MTV contouring, and neck nodes were included only when positive at PET_pre_. In this regard, ATA contouring method was previously developed and validated on pre-clinical data by Brambilla et al. [8, 12]. ATA is a standardized image segmentation tool using an objective function that generates a threshold based upon the characteristics of each tumor lesion and the tumor to background signal ratio thus the pixels are labelled as target (MTV) if their intensity is higher than the threshold, whereas those with a lower intensity are considered non-target). In case of centrally necrotic tumors, the algorithm considers the tissue with [^18^F] FDG-PET uptake surrounding the necrotic area.

**Fig. 1:**
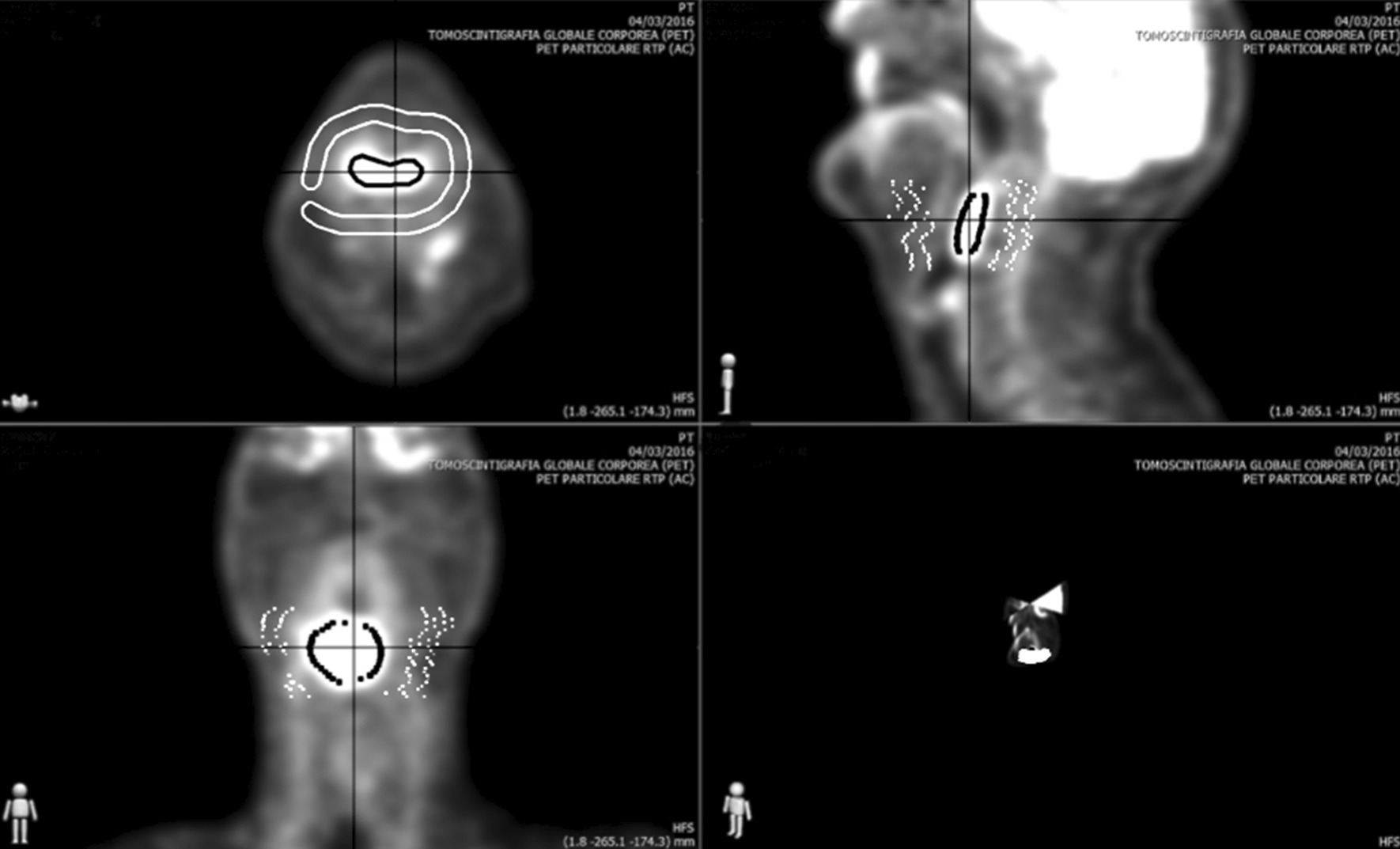
Example of MTV definition on iTaRT workstation of an hypoharyngeal carcinoma in transverse, sagittal and coronal plans and 3D. White contour: VOI_bkg_. Black contour: MTV-PET

PET images were sent to the dedicate iTaRT workstation (Tecnologie Avanzate, Italy). A radiation oncologist expert in the field of HNC defined, the Volumes of Interest (VOIs) surrounding the noticeable FDG uptake for each slice, required to compute the background value (VOI_bkg_) on each PET scan. Once the VOI_bkg_ was drawn, the algorithm automatically defined the background uptake of each patient and produced the adaptive thresholding MTVs (MTV_pre_ and MTV_rec_) in the three directions (transverse, sagittal and coronal) (Fig. 1).

### Data analysis and statistics

Both MTV_pre_ and MTV_rec_ were mapped to the original planning CT images by means of the deformable image registration (DIR) provided by the Ray-Station (RaySearch Laboratories, Sweden) treatment planning system (TPS).

The overlapping volume of MTV_pre_ and MTV_rec_ (MTV_pre&rec_) was delineated automatically by the Boolean Algorithm Tools provided by the TPS.

The percentage of MTV_rec_ inside the MTV_pre_ was adopted as index to rate the inclusion of the recurrence inside the pre-treatment PET/CT uptake. It was calculated normalizing the overlapping volume between the two MTVs to MTV_rec_: the values ranged from 100, when MTV_rec_ is totally included in the MTV_pre_, to 0 when the two volumes are completely disjoint.

The dose coverage of both volumes was estimated on the original planning CT by means of the isodose lines and the dose delivered to the 99% of the volume (D99). The D99 of MTV_pre&rec_ was calculated to state if there were dose “cold spots” within this sub-volume from which recurrence could originate,

The D99 and the D95 (the dose delivered to the 95% of the volume) within MTV_rec_ were calculated to assess the dose coverage of the volume of relapse during RT.

Dose coverage regions were defined as: “high-dose coverage region” if D99 was ≥ 95% of the prescribed dose (≥ 66.5 Gy), “high-dose gradient region” if D99 was < 95% and D95 ≥ 95% of the prescribed dose, “low-dose gradient region” otherwise.

Therefore. the recurrent volume was defined as: ‘‘In-Field (IF)’’ if it received at least > 95% of the prescribed dose, ‘‘Marginal Recurrence (MR)’’ if it extends in the high dose gradient region, or ‘‘Out-of-Field (OF)’’ if it was located in the low-dose gradient region [13].

In this study, we focused on disease failure at the site of FDG uptake area at PET_pre._

## Results

We analyzed 87 out of 126 consecutive patients with squamous HNC treated by IMRT, who met the selection criteria.

At median follow-up of 26 months (22–78 months), 10/87 patients (11%) experienced failure at the site of the primary tumor, detected by clinical examination and imaging studies including CT and/or MRI, PET/CT, and confirmed by biopsy. The median time at recurrence is 16.2 months (4–32 months). One patient had also nodal recurrence. Five other patients had only nodal recurrence and were not included in the current analysis. Patient characteristics at diagnosis are reported in Table 1.

**Table 1:** Characteristics at diagnosis of the 10 patients with T-local recurrence

Features		Value N (%)
Age	Median	66
	Range	38–71
Gender	Male	8 (80%)
	Female	2 (20%)
KPS score	Median	90
	Range	70–100
Smoking	Yes	6 (60%)
	No	4 (40%)
Alcohol	Yes	6 (60%)
	No	4 (40%)
Primary tumour location	Oral cavity	2 (20%)
	Oropharynx	2 (20%)
	Hypopharynx	3 (30%)
	Larynx	1 (10%)
	Nasopharynx	2 (20%)
Grading	G1–2	5 (50%)
	G3–4	5 (50%)
T stage	cT1	1 (10%)
	cT2	2 (20%)
	cT3	7 (70%)
N stage	cN0	2 (20%)
	cN1	1 (10%)
	cN2b	6 (60%)
	cN2c	1 (10%)
AJCC stage	I	0 (0%)
	II	0 (0%)
	III	4 (40%)
	IV	6 (60%)

All the 10 patients with local recurrence had received a full RT dose of 70 Gy. Seven patients had been treated with IMRT-SIB and three with volumetric modulated arc therapy with simultaneous integrated boost (VMAT-SIB).

Five patients (50%) had received also concurrent platinum-based chemotherapy. The other 5 patients had not received chemotherapy because of old age and/or severe comorbidities.

After recurrence detection, five patients received salvage surgery, one of them by endoscopy followed by stereotactic RT with platinum-based chemotherapy, and four patients received only platinum-based chemotherapy or cetuximab. One patient refused any further treatment.

### Pattern of recurrence

The 10 recurrences at primary site had the following characteristics.

Mean GTV-T (based on clinical examination, CT and/or MRI and visual contouring of pre-treatment [^18^F] FDG-PET in treatment position) was 12.7 cc (7.3–42.8 cc; SD = 7.3 cc).

Mean MTV_pre_ derived from ATA was 12.2 cc (4.6–28.9 cc; SD = 7.9 cc) and mean MTV_rec_ derived from ATA was 4.3 cc (1.1–12.7 cc; SD = 4 cc). In all cases the GTV-T resulted greater than MTV_pre_ that was systematically larger than the MTV_rec_.

Mean MTV_pre_ SUV_max_ and SUV_mean_ were 13.3 (7.5–25.4), and 7.9 (3.2–14.7) respectively, while mean MTV_rec_ SUV_max_ and SUV_mean_ were 11.5 (6.9–19.7), and 4.6 respectively (3.3–7.6).

Tumor volumes (GTV-T, MTV_pre_, MTV_rec_ and MTV_pre&rec_) of the 10 patients are summarized in Table 2.

**Table 2:** Tumour volumes of the 10 patients with T-local recurrence

Patients	GTV-T (cm^3^)	MTV_pre_ (cm^3^)	MTV_rec_ (cm^3^)	MTV_pre&rec_ (cm^3^)
1	9.9	6.6	1.2	0.1
2	21.2	19.5	12.7	7.8
3	11.8	9.8	1.4	1.3
4	14.8	10.0	5.5	4.2
5	40.04	19.0	1.5	1.3
6	7.2	4.7	2.1	0.7
7	9.9	4.6	1.1	1.1
8	11.4	7.7	6.1	0.9
9	14.4	11.3	9.3	1.1
10	42.9	28.9	2.3	2.3

GTV-T: encompassing the primary tumour defined using CT, MRI, clinical examination and PET/CT. MTV_pre_: the primary tumour contoured by means of ATA on pre-treatment PET/CT. MTV_rec_: the recurrence contoured by means of of ATA on post treatment PET/CT. MTV_pre&rec_: the overlapping volume of MTV_pre_ and MTV_rec_

*GTV* gross tumor volume, *CT* computer tomography, *MRI* magnetic resonance imaging, *PET* positron emission tomography, *MTV* metabolic target volume, *ATA* adaptive threshold algorithm

In two out of 10 patients, failures were completely (100%) located inside the PET volume defined as MTV_pre_. Four out to 10 recurrences were mostly included (61–91%) in the MTV_pre_, and 4/10 were marginal with respect to MTV_pre_ (1–33%).

**Fig. 2:**
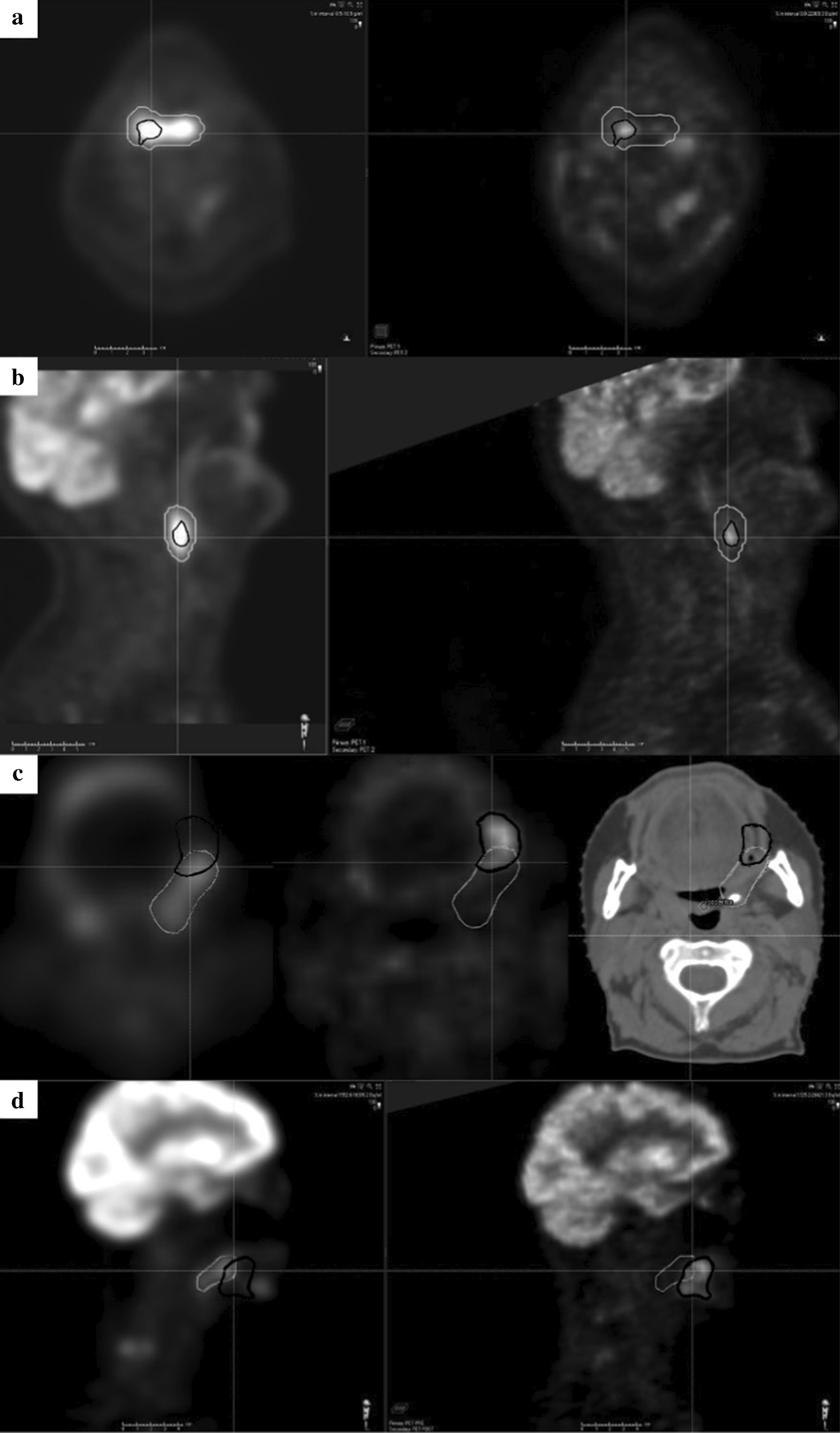
Example of relation between MTV_pre_ and MTV_rec_. Hypopharyngeal recurrence: MTV_rec_ completely inside MTV_pre_. **a** Transverse scans: on the left: PET/CT_pre_. On the right: PET/CT_rec_. **b** Sagittal scans: on the left: PET/CT_pre_. On the right: PET/CT_rec_. Oral cavity recurrence: MTV_rec_ marginal towards MTV_pre_. **c** Transverse scans. On the left: PET/CT_pre_. Centre: PET/CT_post_. On the right Simulation CT. Blue contour: MTV_pre_. Red contour: MTV_rec_. **d** Sagittal scans. On the left: PET/CT_pre_. Centre: PET/CT_rec_. Light gray contour: MTV_pre_. Black contour: MTV_rec_

Examples of relation between MTV_pre_ and MTV_rec_ are shown in Fig. 2.

Mean MTV_pre&rec_ was 2.1 cc (0.1–7.8 cc).

**Fig. 3:**
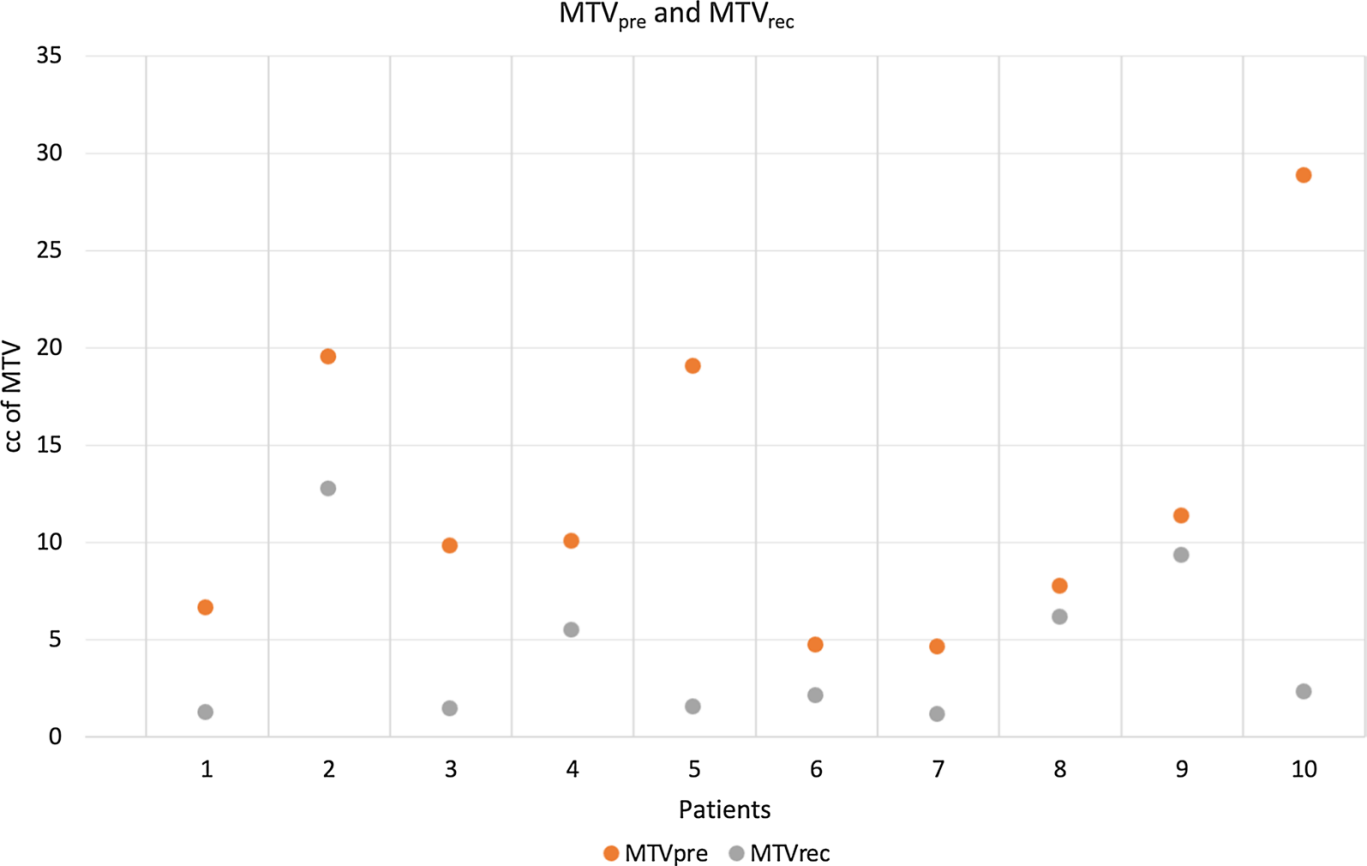
MTV_pre_ and MTV_rec_ for each patient

The volumes of MTV_pre_ and MTV_rec_ of each patient are plotted in Fig. 3.

Mean D99 of MTV_pre&rec_ was 67.9 Gy (66.2–69.2 Gy) considering a prescription dose of 70 Gy to the PTV.

In five out of 10 patients, recurrence volume was included in high-dose coverage region (D99 between 66.5 and 68.7 Gy).

Four out of the remaining 5 recurrences were inside the high gradient of dose region: two were nearly overlapped with the high dose coverage region (D99 = 64.8 Gy and D99 = 66.4 Gy); two extended in the dose falloff region below the 90% of the prescribed dose (D99 = 54.7 and D99 = 61.7 Gy). A single recurrence was found in the lower dose gradient region (D99 = 44.6 Gy, D95 = 47.4).

Thus, recurrences were classified as follows: five (50%) as “in field” (IF), 4 (40%) as “marginal recurrence” and one (10%) as “out of field” (OF).

**Fig. 4:**
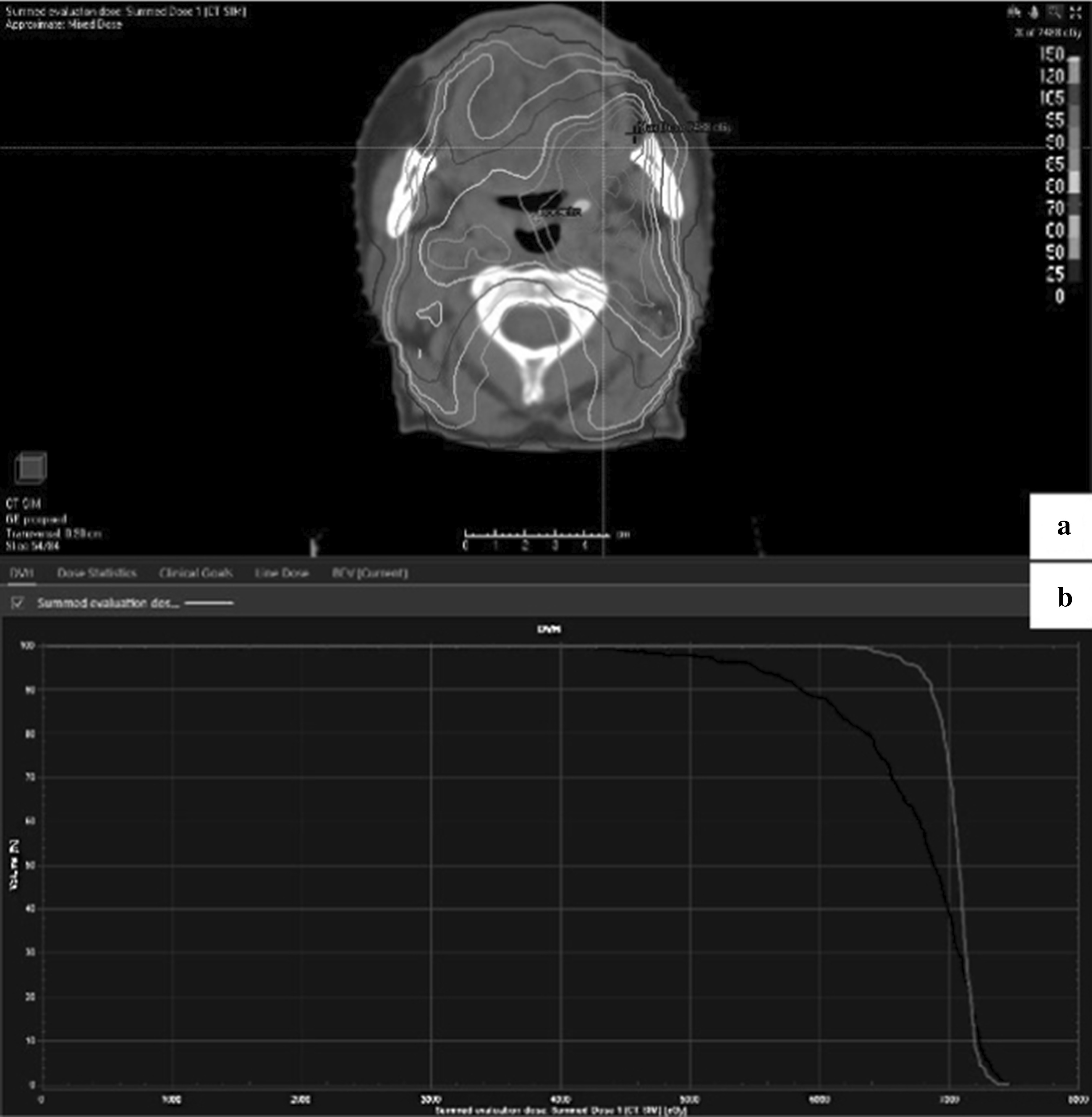
Example of oral cavity recurrence “out of field”. **a** Planning CT displayed in transverse plans with isodose lines. **b** Dose-volume histograms (DVH) showing an appropriate coverage of MTV_pre_. MTV_rec_ is outside dose coverage region (D99 = 44.7 Gy and MTV_rec_ D95 = 47.4 Gy). Light gray contour: MTV_pre_. Black contour: MTV_rec_

An example of out of “out of field” recurrence is shown in Fig. 4.

## Discussion

Recurrence at the primary tumor site occurs approximately in 20–50% of patients with HNC and remains the most common cause of treatment failure [4, 14]. Post treatment assessment is crucial because further treatments depend on it [15], but an accurate restaging could be a challenging issue due to therapies which can cause relevant tissue changes related to inflammation/fibrosis and consequently distortion of the anatomy.

In this setting, PET/CT is superior to morphological imaging in the assessment of tumor response and detection of recurrence, as demonstrated by two meta-analyses [16, 17]. Moreover, a recent systematic review showed that MTV and TLG could be useful for identifying patients with a higher risk of postsurgical disease progression who could receive early therapeutic intervention to improve their prognosis [18].

Many studies showed that failures are often within pre-treatment PET/CT volume. Madani et al. in their series found that most of the local relapses occurred inside the pre-treatment MTV or at its borders (5/9 i.e. 56% of the patients) [19]. La et al. found 6/7 (86%) local failures within the pre-treatment MTV [10] and Soto et al. found a higher rate of local failure within the pre-treatment MTV (8/9 patients, i.e. 89%) [9].

Our findings are consistent with these data: failures are completely located in MTV_pre_ in 2/10 patients (20%) and they were mostly inside (61–91%) included in MTV_pre_ (total: 6/10 recurrence within MTV_pre_) in 4/10 patients (40%). In the latter cases, we could argue that the onset of recurrence was primarily inside the MTV_pre_ and then extended also outside.

Therefore, PET volumes may encompass radio-resistant areas that warrant more aggressive therapy [20].

Daisne et al. examined the feasibility of target volume delineation based on the PET/CT uptake and found that, even if PET/CT was the imaging modality that better depicted the real extent of the tumor, it failed to detect a small fraction of the macroscopic tumor extension as CT and MRI [6]. This finding may explain the occurrence of local recurrence outside the MTV: Soto et al. observed 1/9 (11%) local failure outside the Biological Target Volume (BTV)[9] and Madani et al. reported 3/9 (33%) local failure outside the BTV [19].

In our series, 4 recurrences (40%) were marginal to MTV_pre_ (< 50% of recurrence volume) and three of them were included for less than 15% in MTV_pre_. In these cases, we could consider that recurrence primarily occurred outside the MTV_pre_ meaning that it could not be fully representative of the real tumor extension.

Madani’s and Soto’s series, and our study as well, are likely to underestimate the true risk of failures outside the MTV because since treatment purpose GTV was defined not only on PET/CT uptake, but also on clinical examination, CT and MRI images [9, 19].

Thus, PET/CT should not be used as a single imaging modality in target volume definition, but it could be used to identify pre-treatment potentially more aggressive or resistant sub-volumes within the tumor.

Madani et al. reported good results in dose escalating radiation dose on PET avid region within the target volume both in terms of complete response (complete response in 81–86% of patients), 1-year local control (85–87%) and 1-year overall survival (54–82%) [19].

PET defined target volume (MTV) is smaller than CT based counterpart (GTV-CT), but the additional information obtained from PET can enlarge the GTV-CT in 25% of cases and this percentage can increase to up to 64% depending on the SUV threshold adopted [21]. These results confirm the value of using PET/CT as an additional source of information to achieve an adequate target coverage.

Half patients of our series received concurrent cisplatin-based chemotherapy. The two patients with the local recurrence entirely inside the MTV_pre_ did not receive chemotherapy because of comorbidity. In such patients, an intensification of radiotherapy treatment might be a way to improve the therapeutic ratio.

In all the 10 patients, an overlap MTV_pre&rec_ existed between the planning PET_pre_ and the PET_rec_, which indicates a high probability that recurrence could originate from the MTV_pre_.

The mean dose received by the 99% of the MTV_pre&rec_ volume was 67.9 Gy, (66.2–69.2 Gy): considering the prescription dose of 70 Gy to the PTV, dose coverage of these volumes was optimal. It is not possible to determine if higher radiation doses to PET/CT based sub-volumes could affect failure rates and outcomes. Our data supports the need to explore this hypothesis because most of failures were mapped inside the MTV_pre_.

In this regard, Thorwarth et al. published a study on dose painting by numbers (DPBN) in thirteen HNSCC patients exploring the use of PET/CT in identifying radioresistant areas within the tumor [22]. For each patient, a conventional IMRT plan, a plan with an additional uniform dose escalation of 10% to the FDG volume (uniDE) and a plan with DPBN were obtained and analyzed according to a map calculated from FMISO-PET. Both dose-escalation approaches were shown to be feasible under the constraint of limiting normal tissue doses to the level of conventional IMRT and for DPBN; the prescription could be fulfilled in larger regions of the target by using DPBN rather than uniDE. Tumor control probability increased from 55.9% with conventional IMRT to 57.7% for the uniDE method. For DPBN a potential increase in tumor control probability from 55.9 to 70.2% was calculated [22].

In our study, recurrences were classified as IF in 5 cases (50%), as marginal recurrence in four cases (40%) and as OF in one case (10%). The 5 IF failures, documented by the (MTV)_pre&rec_, were included in high-dose coverage region (D99 between 66.4 and 68.7 Gy) or inside high gradient of dose region (D99 = 64.8 Gy and D99 = 66.4 Gy); the four marginal failures were partially included in high dose coverage and high gradient of dose region and the one OF failure was outside high dose coverage region (D99 = 44.7 Gy and D95 = 55.1 Gy).

Our study suggests that patients with IF failure could benefit of a higher dose to achieve local control, and patients with marginal failure could benefit of an improved dose coverage, by an optimization of GTV and CTV delineation. Besides integration of different imaging and clinical data, PET/CT based contouring could also be improved. As shown in Brambilla’s study, radiation oncologists should probably move from visual PET interpretation to an established method for PET volume segmentation and reconstruction algorithms [12].

There are some limitations in our study. As reported also in the other literature series, a relatively small number of treatment failures was observed and analysed, limiting the possibility to perform a statistical analysis. Another concern is that failures were heterogeneous with respect to tumor location and stage. Given the number of subjects included, our results should be verified in larger series.

On the other hand, all patients were treated in a very homogeneous way and all PET imaging studies were performed with similar technical characteristics by the same scanner with the patient in treatment position.

## Conclusion

The present study was able to map local recurrence in HNC patients in relation to MTV detected by [^18^F] FDG-PET, providing an accurate identification of dose levels in terms of D99 and D95 of the metabolic recurrent volume (MTV_rec_) also in relation to the pre-treatment assessment (MTV_pre_).

Although MTV_pre_ had an appropriate dose coverage, i.e. there were no under-dosed regions within the target volumes, 50% of patients had “in field” (IF) recurrence whereas 40% of patients experienced a marginal recurrence in the “dose gradient area” (Table 3).

**Table 3:** Pattern of failure of the 10 patients with T-local recurrence

Patients	D99 MTV_pre&rec_ (Gy)	D99 MTV_rec_ (Gy)	D95 MTV_rec_ (Gy)
1	69.2	54.7	67.1
2	68.1	61.7	67.6
3	67.9	67.8	68.1
4	66.7	67.0	68
5	66.2	65.2	66.6
6	68.7	68.7	69.1
7	66.5	66.5	67.6
8	68.1	44.7	47.4
9	68.5	64.8	66.5
10	68.6	68.5	68.8

D99 MTV_pre&rec_: the dose delivered to the 99% of the overlapping volume of MTV_pre_ and MTV_rec_. D99 MTV_rec_: the dose delivered to the 99% of the volume of MTV_rec_. D95 MTV_rec_: the dose delivered to the 95% of the volume of MTV_rec_

*MTV* metabolictarget volume

In the first case, a dose intensification could be useful to decrease the risk of failure, while in the second case a careful optimization of the target volume in terms of GTV and CTV, including MTV, with suitable margins could be useful to reduce the recurrence rate. Dose intensification is a hypothesis that could be investigated in further studies.

## Data Availability

The datasets analyzed during the current study are available from the corresponding author on reasonable request.

## Abbreviations

HNSCC: Head and neck squamous cell carcinoma
HNC: Head and neck cancers
RT-CHT: Radio-chemotherapy
IMRT-SIB: Intensity modulated radiation therapy with simultaneous integrated boost
RT: Radiotherapy
MTV: Metabolic target volume
BTV: Biological target volume
IF: In-field
PTV: Planning target volume
IMRT: Intensity modulated radiation therapy
GTV: Gross target volume
HR-CTV: High risk clinical target volume
LR-CTV: Low risk clinical target volume
ATA: Adaptive threshold algorithm
VOI: Volumes of interest
TPS: Treatment planning system
DIR: Deformable image registration
VMAT-SIB: Volumetric modulated arc therapy with simultaneous integrated boost
DPBN: Dose painting by numbers
KPS: Karnofsky Performance Score
AJCC: American Joint Committee on Cancer
